# Methylation class oligosarcoma may encompass IDH-wildtype gliomas

**DOI:** 10.1007/s00401-022-02529-x

**Published:** 2022-12-15

**Authors:** Azadeh Ebrahimi, Ulrich Herrlinger, Andreas Waha, Luisa Kaluza, Torsten Pietsch

**Affiliations:** 1 Department of Neuropathology, University of Bonn, Venusberg Campus 1, 53127 Bonn, Germany; 2 Division of Clinical Neuro-Oncology, Department of Neurology, University of Bonn, Venusberg Campus 1, 53105 Bonn, Germany

Oligosarcoma was recently introduced as a distinct group within the family of *IDH *mutant gliomas [9]. The diagnosis requires the combined presence of (a) sarcomatous histology and (b) *IDH *mutation and (c) *TERT* promoter mutation and/or 1p/19q codeletion. In unresolved cases, a characteristic DNA methylation profile is suggested to be diagnostic [9].

We identified a rare recurrent case of adult diffuse glioma, *IDH*-wildtype in a 63-year-old female patient, showing a long clinical course and falling into the recently defined methylation class oligosarcoma, *IDH *mutant [9], but lacking an IDH mutation. Besides, the tumor showed multiple copy number alterations including gain of chromosome 7, loss of chromosome 10 and homozygous deletion of *CDKN2A/B,* typically seen in glioblastoma/ gliosarcoma, *IDH*-wildtype.

The patient had primarily presented with a right frontal lesion in 1988. She had initially received a seed implantation for an unknown duration that was eventually removed, and the tumor was resected and diagnosed as a diffuse astrocytoma. The follow-up imaging studies until 2005 did not show any recurrence. In July 2021, the patient started to experience new neurological signs and symptoms including hemiparesis. The neuroradiological examinations revealed a contrast-enhancing multi-lobulated and cystic appearing tumor within the previous resection cavity, with extension to the basal ganglia and infiltration of cortex (Fig. 1a, b). The tumor was resected and histological examination revealed a poorly differentiated malignant tumor with an extensive sarcomatous histology, areas of palisading necrosis, microvascular proliferations and abundant mitoses (Fig. 1c–f). Similar to previously defined cases of oligosarcoma [9], the tumor lacked Olig2 and GFAP but showed expression of MAP2c and widespread expression of oncogenic YAP1. The expression of H3K27me3 was significantly reduced but not completely lost (Fig. 1g–j). The nuclear expression of ATRX was retained. Immunohistochemical staining with antibody against mutation-specific IDH (R132H) protein was negative. Immunohistochemical expressions of mismatch repair proteins MSH2, MHS6, MLH1 and PMS2 were retained.

**Fig. 1:**
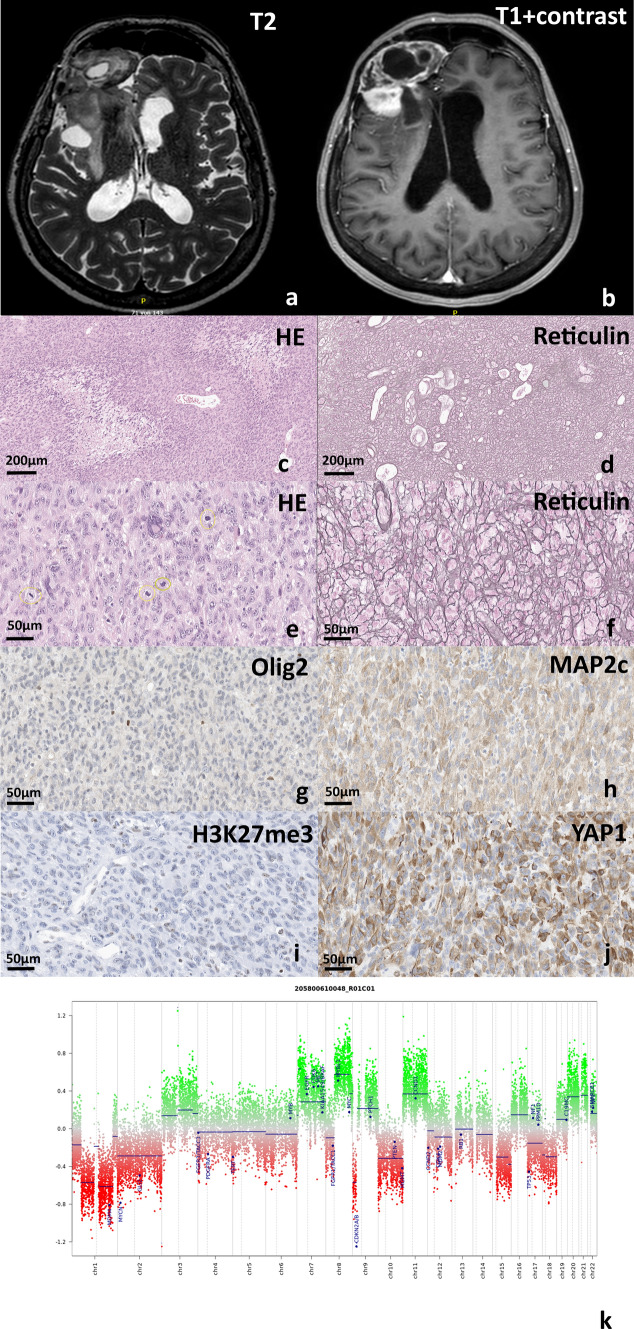
MRI showed a contrast-enhancing multi-lobulated and cystic appearing tumor within the previous resection cavity (**a**, **b**). The tumor revealed a diffuse sarcomatous histo-morphology with areas of palisading necrosis (**c**) and endothelial proliferations (**d**), many mitotic figures (**e**), abundant reticulin (**f**), lack of Olig2 expression (**g**) but expression of MAP2c (**h**), reduced H3K27me3 expression (**i**), and extensive expression of oncogenic YAP1 (**j**). Copy number profile of the tumor revealed multiple chromosomal alterations also including typical constellation + 7/−10 and homozygous deletion of *CDKN2A/B* usually present in glioblastoma/ gliosarcoma, IDH-wildtype (**k**)

The next-generation sequencing on a MiSeq sequencer was performed using 200 ng FFPE DNA, and the AmpliSeq^®^ for Illumina targeted resequencing technology with 1074 custom amplicons (AmpliSeq for Illumina Custom Glioma Panel). The glioma panel contains the entire coding regions of the genes *ACVR1, ATRX, CDKN2A, CIC, EGFR, ERBB2, FUBP1, MLH1, MSH2, MSH6, NF1, PDGFRA, PIK3CA, PIK3R1, PMS2, POLD1, POLE, PPM1D, PTEN, RB1, TP53, GNA11, GNAQ, HRAS, NRAS, MYB, MYBL1, MYC, MYCN, NF2, NRAS and SETD2* in addition to the defined regions that represent established mutation hotspots in gliomas; these comprise codons of *IDH1* (codon 132), *IDH2* (codon 172), *H3F3A* (codons 27 and 34), *TERT *promoter positions (C228T c.− 124C > T Chr.5:1295228C > T, C250T c.− 146C > T Chr.5:1295250C > T), *Hist1H3B* (codon 27), *Hist1H3C* (codon 27), *BRAF* (codon 600) and *FGFR1* (codons 546, 655 and 656). The analysis revealed a hotspot *pTERT* mutation (NM_198253.2:c.− 124C > T), a nonsense/stop mutation in *TP53* (NM_000546.5:c.310C > T) and a *CIC* mutation (NM_015125.3:c.4543C > T), but no *IDH* mutations. The *CIC* mutation was previously reported in an oligodendroglioma [2], knowing that *CIC* mutations are found in glioblastomas too [10]. A control pyrosequencing of *IDH1* (codon 132), *IDH2* (codon 172) confirmed the wildtype status, and additional TSO500 (Illumina) sequencing confirmed these results. TSO500 panel covers the coding sequence of 523 cancer-associated genes. All genes listed in the custom glioma panel are included in the TSO500 panel. RNA sequencing using TruSight fusion panel (Illumina) did not show any in-frame fusions. Typical mutations and numerical alterations of the genes with established relevance for glioblastoma/gliosarcoma, IDH-wildtype comprising *CCND1, CCND2, CDK4, CDK6, EGFR, MDM4, MET, NF1, PDGFRA, PTEN* and *RB1* were not observed.

DNA methylation profile generated via the Infinium MethylationEPIC (850 k) BeadChip array (Illumina, San Diego, USA) showed a significant calibrated score of 0.96 for methylation class oligosarcoma, *IDH *mutant in version 12.5 of brain tumor classifier and no match (all scores lower than 0.3) in version 11b4 of the classifier (Fig. 1k). *MGMT* promotor was only slightly methylated (MGMT-STP27).

To date, few cases of oligosarcoma have been reported, some of which date back to the pre-*IDH* era [1, 3, 4, 7–9, 11, 12]. Most of these cases arise from primary oligodendroglioma. In the recently published cohort of methylation class oligosarcoma, 12 oligosarcomas were presented as primary manifestations [9]. We could not retrieve the primary tumor material of our patient to reevaluate the diagnosis of 1988. As yet, all reported cases, which were examined for *IDH* status, harbored one of the known hotspot *IDH* mutations, though 1p/19q codeletion was not always present [7]. Oligosarcoma presents a characteristic methylation profile [9]. Our case showed multiple chromosomal gains and losses including a gain of chromosome 7, loss of chromosome 10 and *CDKN2A/B* homozygous deletion*,* a constellation typically present in glioblastoma/gliosarcoma, *IDH*-wildtype. However, considering the high copy number variation in the tumor, the specificity of this constellation here might be questionable. Focal loss of *IDH* mutation within a primary IDH-mutant tumor has been reported as a rare event after radiotherapy and due to a loss of heterozygosity [6]. Similarly, a monosomy of chromosome 2 and 15 harboring *IDH1* and *IDH2* genes might have caused a loss of mutant allele in the present case too. Most of the reported oligosarcomas have a history of radiation therapy. The time to recurrence can be very variable from about 1 year to more than 30 years as in our case [9]. Radiation-induced glioblastoma/gliosarcoma is a differential diagnosis in this case. However, radiation-induced glioblastoma/gliosarcoma mostly lacks a 7 + /10− signature and *pTERT* hotspot mutations [5], which are typically present in the vast majority of spontaneous adult age IDH-wildtype glioblastomas/gliosarcoma. We believe that this case might add useful information to the newly defined methylation class oligosarcoma, and* IDH *mutant expands the spectrum of cases included in this tumor class.

## Data Availability

The datasets generated and/or analysed during the current study are available from the corresponding author on reasonable request.
